# Ebola Virus Glycoprotein Induces an Innate Immune Response *In vivo* via TLR4

**DOI:** 10.3389/fmicb.2017.01571

**Published:** 2017-08-17

**Authors:** Chih-Yun Lai, Daniel P. Strange, Teri Ann S. Wong, Axel T. Lehrer, Saguna Verma

**Affiliations:** Department of Tropical Medicine, Medical Microbiology and Pharmacology, John A Burns School of Medicine, University of Hawaii at Manoa Honolulu, HI, United States

**Keywords:** Ebola virus, glycoprotein, innate immunity, TLR4, Ebola vaccine

## Abstract

Ebola virus (EBOV), a member of the *Filoviridae* family, causes the most severe form of viral hemorrhagic fever. Although no FDA licensed vaccine or treatment against Ebola virus disease (EVD) is currently available, Ebola virus glycoprotein (GP) is the major antigen used in all candidate Ebola vaccines. Recent reports of protection as quickly as within 6 days of administration of the rVSV-based vaccine expressing EBOV GP before robust humoral responses were generated suggests that the innate immune responses elicited early after vaccination may contribute to the protection. However, the innate immune responses induced by EBOV GP in the absence of viral vectors or adjuvants have not been fully characterized *in vivo*. Our recent studies demonstrated that immunization with highly purified recombinant GP in the absence of adjuvants induced a robust IgG response and partial protection against EBOV infection suggesting that GP alone can induce protective immunity. In this study we investigated the early immune response to purified EBOV GP alone *in vitro* and *in vivo*. We show that GP was efficiently internalized by antigen presenting cells and subsequently induced production of key inflammatory cytokines. *In vivo*, immunization of mice with EBOV GP triggered the production of key Th1 and Th2 innate immune cytokines and chemokines, which directly governed the recruitment of CD11b^+^ macrophages and CD11c^+^ dendritic cells to the draining lymph nodes (DLNs). Pre-treatment of mice with a TLR4 antagonist inhibited GP-induced cytokine production and recruitment of immune cells to the DLN. EBOV GP also upregulated the expression of costimulatory molecules in bone marrow derived macrophages suggesting its ability to enhance APC stimulatory capacity, which is critical for the induction of effective antigen-specific adaptive immunity. Collectively, these results provide the first *in vivo* evidence that early innate immune responses to EBOV GP are mediated via the TLR4 pathway and are able to modulate the innate-adaptive interface. These mechanistic insights into the adjuvant-like property of EBOV GP may help to develop a better understanding of how optimal prophylactic efficacy of EBOV vaccines can be achieved as well as further explore the potential post-exposure use of vaccines to prevent filoviral disease.

## Introduction

The recent outbreak of Ebola virus (EBOV, also known as Zaire Ebola virus) in several West African countries in 2013–16 is by far the largest and most complex filovirus outbreak and has brought the virus and Ebola virus disease (EVD) to the forefront of interest as an emerging infectious disease. The World Health Organization has reported 28,616 confirmed and suspected cases and 11,310 deaths (World Health Organization, 2016). The quick spread of EBOV infection outside the outbreak regions into other African countries such as Nigeria and Mali, and the United States indicates that EBOV has become a global threat to public health and uncertainty exists regarding future outbreaks of EBOV and other filoviruses such as Marburg virus (MARV) (Breakwell et al., 2016).

EBOV is a member of the *Filoviridae* family and is classified in the genus *Ebolavirus*, species *Zaire ebolavirus*. It causes severe disease and high case fatality rates in humans (Feldmann et al., 2013). The single stranded, negative-sense RNA genome of EBOV encodes seven viral structural proteins including nucleoprotein (NP), and virion protein (VP) 35, VP40, glycoprotein (GP), VP30, VP24, and RNA-dependent RNA polymerase (L) (Feldmann et al., 2013). The open reading frame (ORF) coding for EBOV GP also gives rise to non-structural soluble GP (sGP) and shed GP, which is generated from the mature trimeric surface GP via proteolytic cleavage of the transmembrane region by TACE (TNF-α converting enzyme) (Dolnik et al., 2004), and released from infected cells (Sanchez et al., 1996; Dolnik et al., 2004). GP is the only viral protein exposed on the surface of mature viral particles and associated with induction of protective immune responses.

EBOV initially targets mononuclear phagocytic cells (monocytes and macrophages) and dendritic cells (DCs) that play a critical role in virus dissemination and spread to the liver, spleen and other tissues and cell types (Zaki et al., 1999; Geisbert et al., 2003b,a). Studies of non-human primates (NHPs) and *in vitro* models showed that EBOV infection triggers monocytes and macrophages to induce strong innate immune responses including production of several inflammatory cytokines and chemokines such as interleukin (IL)-1β and IL-6, and tumor necrosis factor (TNF) (Gupta et al., 2001; Stroher et al., 2001; Hensley et al., 2002; Wahl-Jensen et al., 2011), but fails to activate DCs (Mahanty et al., 2003; Lubaki et al., 2013). Previous *in vitro* studies using virus-like particles (VLPs) have demonstrated that macrophages and DCs can be activated by GP and produce cytokine and chemokines through the TLR4 signaling pathway, which further supports T cell proliferation (Bosio et al., 2004; Wahl-Jensen et al., 2005; Okumura et al., 2010; Ayithan et al., 2014). A recent report showed that shed GP activates macrophages and DCs, which may cause a massive release of pro- and anti-inflammatory cytokines and affect vascular permeability (Escudero-Perez et al., 2014). In addition, EBOV or GP was shown to enhance monocyte maturation, which promote virus infection, further causing the death of T lymphocytes (Iampietro et al., 2017). However, GP-induced innate immune responses have not been fully characterized *in vivo*.

There is currently no FDA approved antiviral therapy or vaccine available for prevention of EVD and treatment is limited to supportive care. While cocktails of monoclonal antibodies as well as antivirals have been tested as experimental therapies mainly during the recent West African outbreak, a safe and efficacious vaccine is still the most economic and effective countermeasure to prevent large-scale filovirus outbreaks (Dye et al., 2012; Pettitt et al., 2013; van Griensven et al., 2016; Trad et al., 2017). Several vaccine approaches including virally-vectored vaccines such as recombinant vesicular stomatitis virus (rVSV), recombinant adenoviruses, and protein-based subunit vaccines such as virus-like particles (VLPs) have been demonstrated to protect against filovirus infection in both small animal models such as mice as well as in non-human primates (NHPs) (Ohimain, 2016). EBOV GP is the prime target for developing protective antibodies, therefore all Ebola vaccine candidates have GP as a key (and often the only) antigen. The results from the recent human EBOV vaccine clinical trial in Guinea are encouraging and indicate that rVSV-ZEBOV is safe and highly efficacious in preventing EVD when delivered via a ring vaccination strategy during an outbreak (Henao-Restrepo et al., 2015, 2017). However, despite active research on EBOV vaccines, the specific mechanisms by which GP mediates immune protection are not yet fully understood.

The results from the phase 3 vaccination trial using rVSV-ZEBOV showed that this vaccine induced protection as quickly as 6 days after administration even before robust IgG responses were generated (Henao-Restrepo et al., 2015). Furthermore, the observation that EBOV VLPs containing GP can elicit protection in mice even when given after virus challenge (Ayithan et al., 2015; Bradfute et al., 2015), strongly suggests that an adaptive response may not be the only way by which EBOV GP can confer protective immunity. Based on prior outbreak reports and recent clinical studies, it appears that the difference between EVD survivors and fatalities lies in the early immune responses elicited during the virus infection that may also explain protection in the recently used ring vaccination approach (Leroy et al., 2000; Baize et al., 2002).

Both innate and adaptive immune responses are important for robust vaccine-induced protection. Vaccine adjuvants typically function to boost the innate immune response; therefore, understanding of the specific innate immune response induced by the antigen alone is important to optimize antigen and adjuvant formulations and dosing schedules. In the present study, we investigated whether purified GP alone in the absence of other viral components and adjuvants can activate APCs and induce innate responses including the production of inflammatory cytokines using a murine model. We show here that exposure of mouse bone marrow-derived macrophages (BMDMs) to EBOV GP induced robust production of cytokines TNF-α and IL-1β but not of type I IFN. *In vitro* experiments using BMDMs demonstrated that GP was efficiently internalized by APCs and up-regulated expression of co-stimulatory molecules, suggesting that GP can enhance the stimulatory capacity of APCs, which is critical for induction of effective antigen-specific adaptive immunity. *In vivo*, GP also triggered production of multiple cytokines and chemokines and we further demonstrated that the GP-induced cytokine response occurs via activation of TLR4 signaling, directly affecting the recruitment of immune cells to the draining lymph nodes. Collectively, our data provide the first *in vivo* evidence that GP-induced innate immunity is via TLR4 and modulates key immune events critical for early control of the virus as well as fine tuning the innate-adaptive interface.

## Materials and methods

### Ethics statement

This study was carried out in accordance with the recommendations in the Guide for the Care and Use of Laboratory Animals of the National Institutes of Health. The protocol was approved by the Institutional Animal Care and Use Committee at the University of Hawaii, and all efforts were made to minimize animal suffering.

### Recombinant protein

**Figure 1:**
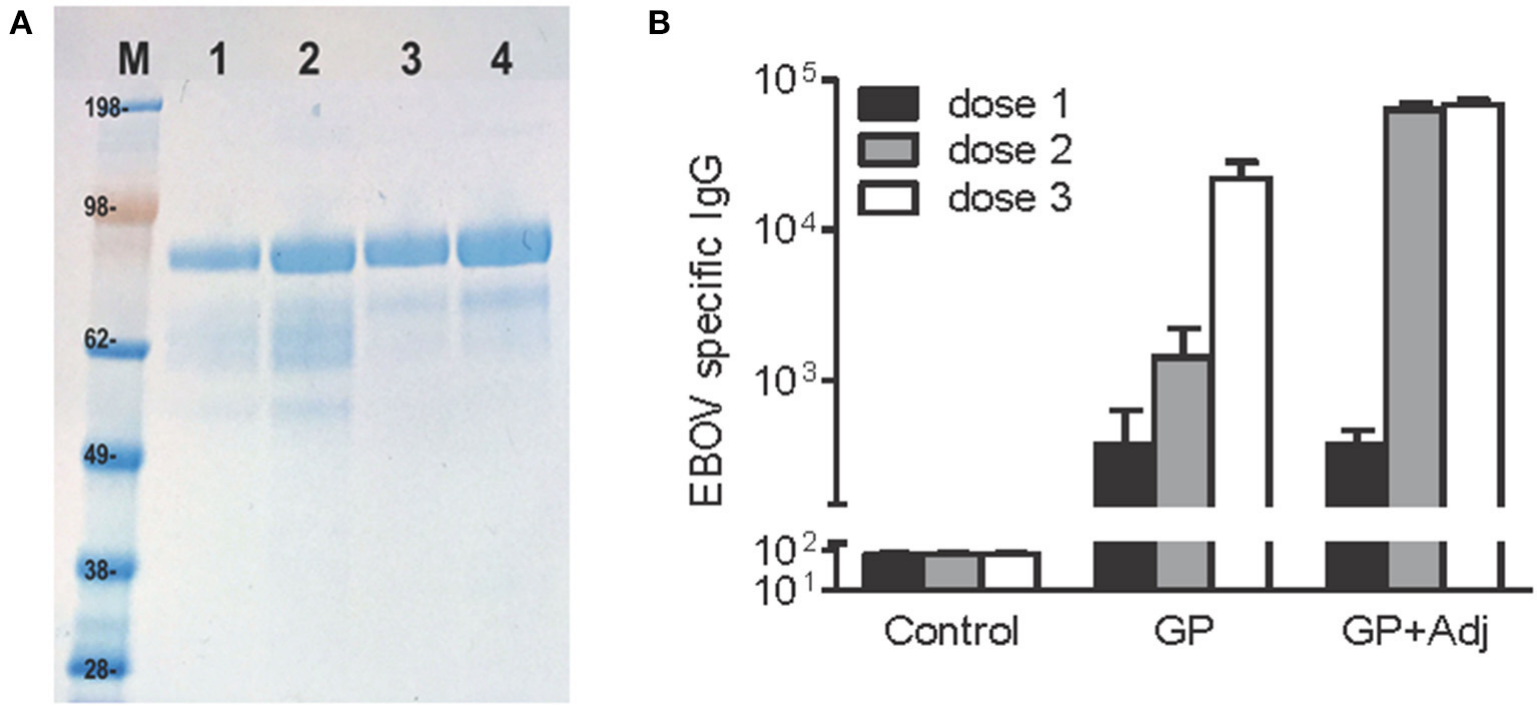
Highly purified, recombinant EBOV GP induces potent antibody responses after two and three doses. **(A)** EBOV GP protein on SDS-PAGE stained using Coomassie blue. M, molecular weight marker, Lanes 1–2, 1 and 2 μg of single-step immunoaffinity purified GP protein (90% purity) and lanes 3–4, 1 and 2 μg of protein after 2-step purification (95% purity). **(B)** BALB/c mice were inoculated with GP (10 μg per dose) via subcutaneous route with or without adjuvant, followed by two booster doses in 4-week intervals. EBOV specific IgG was measured using a standard ELISA reporting endpoint titers (absorption >0.2 above background). Y-axis: GMT+95%CI.

Recombinant EBOV GP was produced from insect cells and purified using immunoaffinity chromatography following previously published methods (Lehrer et al., 2017). To further increase the level of purity, protein used for our studies was subjected to an additional purification step via size-exclusion chromatography using a HiLoad 16/600 Superdex 200 prep grade column (GE Healthcare Life Sciences, Piscataway, NJ) equilibrated in phosphate buffered saline, pH7.4 (Figure 1A). FITC labeling of purified EBOV GP was performed using the Pierce FITC Antibody Labeling Kit (Thermo Fisher Scientific) according to manufacturer's instructions. Briefly, protein prepared in borate buffer (50 mM sodium borate, pH 8.5) was added to FITC reagent and mixed by pipetting up and down. After incubation for 60 min at room temperature, the labeling reaction was added to the spin column containing a purification resin to remove unbound FITC. After thorough mixing, the purified FITC-labeled protein was eluted by centrifugation of the spin columns for 30–45 s at 1,000 g.

### Cell culture

Human THP-1 cells, a monocytic cell line, obtained from the American Type Culture Collection (ATCC) were grown in RPMI culture media (Sigma) containing 10% fetal bovine serum (FBS) (GE Healthcare Life Sciences), 1% penicillin/streptomycin (Pen/Strep) (Invitrogen), and 0.0006% β-mercaptoethanol (Bio-Rad). Mouse bone marrow-derived macrophages (BMDMs) were obtained by differentiation of bone marrow progenitor cells as described previously (Kumar et al., 2013). Briefly, bone marrow cells were isolated from the pelvis, femurs, and tibias of wild type C57BL/6 or BALB/c mice. Red blood cells (RBC) were lysed using BD Pharm Lyse™ lysing solution (BD Biosciences), and the cells were cultured for 7 days in DMEM (Sigma) supplemented with 10% FBS, 1% Pen/Strep, and 14% (v/v) L929-conditioned medium which contains macrophage colony-stimulating factor (M-CSF) secreted by L929 cells (Weischenfeldt and Porse, 2008). Culture medium was replaced on day 3 and 6 of culture, and fully differentiated mouse macrophages were used for experiments on day 7 of culture.

### Analysis of cytokine gene expression *in vitro* by quantitative real-time PCR

THP-1 cells and mouse BMDMs were treated with EBOV GP (1μg/ml), and the cell culture supernatants and cell lysates were harvested at 2, 6, and 24 h after treatment for further analysis. The total cellular mRNA was extracted and reverse transcribed to cDNA using the NucleoSpin® RNA kit (Macherey-Nagel) and iScript cDNA synthesis kit (Bio-Rad). The synthesized cDNA was subjected to qPCR using iQ SYBR Green Supermix (Bio-Rad) and cytokine gene-specific primers as described previously (Verma et al., 2010; Kumar et al., 2013). The fold change of mRNA levels in GP treated cells was calculated compared to mock after normalizing to the GAPDH gene as described previously (Verma et al., 2010; Kumar et al., 2013).

### *In vitro* assay for antigen uptake by mouse BMDMs

BMDMs prepared from C57BL/6 mice were re-plated in a non-tissue culture treated 24-well plate at a density of 5 × 10^5^ cells per well on day 6 of differentiation. Twenty-four hours later, the cells were treated with medium only (control), or 10 μg/ml of GP-FITC. After 30 min of incubation at 37°C, the reaction was stopped by washing with ice cold 1x PBS. The adherent cells were detached by incubation with Cellstripper solution (Corning) at 37°C for 30 min, washed, and fixed with 4% paraformaldehyde in PBS at 4°C for 30 min. The purity of differentiated BMDMs was determined by staining of the cells with APC-conjugated anti-mouse CD11b antibody (clone M1/70) (eBioscience). GP-FITC^+^ or CD11b^+^ cells were evaluated on a FACSCalibur flow cytometer (BD Biosciences). All flow data were analyzed using FlowJo software (TreeStar Inc).

### *In vitro* assay for the expression of costimulatory molecules in mouse BMDMs

Differentiated BMDMs prepared from C57BL/6 mice were re-plated in a non-tissue culture treated 24-well plate at 5.0 × 10^5^ cells per well. The cells were treated with medium only (control), 10 μg/ml purified recombinant EBOV GP, or 100 ng/ml of LPS (Invivogen). On day 2 of treatment, the cells were detached using Cellstripper solution (Corning), washed with cold PBS supplemented with 2% FBS and Fc-blocked by incubation with anti-mouse CD16/CD32 antibody (eBioscience). The cell surface markers CD40 and CD80 were stained using the following antibodies: APC-conjugated anti-mouse CD40 (clone 1C10) and PE-conjugated anti-mouse CD80 (clone 16-10A1) (eBioscience). The purity of differentiated macrophages was confirmed by staining of the cells with FITC-conjugated anti-mouse CD11b antibody (clone M1/70) (eBioscience). After incubation with antibodies, cells were washed, fixed with 4% paraformaldehyde in PBS, and analyzed using a FACSCalibur flow cytometer (BD Bioscience) as described previously (Kumar et al., 2013). Fluorescence minus one (FMO) samples were prepared for each fluorochrome to facilitate gating. The data were analyzed using Flowjo software (Treestar Inc.).

### Mouse experiments

Wild-type C57BL/6, and BALB/c, and Swiss Webster mice were bred in our laboratory using breeding stock obtained from Jackson Laboratories and Taconic Farms, Inc., respectively. This study was specifically approved by the University of Hawaii Institutional Animal Care and Use Committee (IACUC), and conducted in strict accordance with guidelines established by the National Institutes of Health and the University of Hawaii IACUC. Seven to eight-week old mice were administered EBOV GP (100 μg per mouse) intraperitoneally (i.p.). In some experiments, mice were pre-treated with a TLR4 antagonist, ultrapure lipopolysaccharide from the bacterium Rhodobacter sphaeroides (LPS-RS) (InvivoGen) at the dose of 5 and 10 μg per mouse via i.p. route on day −2 and −1 before administration of GP, respectively. A group of mice was also administered an equal amount of total protein (100 μg per mouse) of the cell culture supernatant from Drosophila S2 cells prepared by the same procedure as the GP (NULL control). Sera were collected at 6 and 24 h after GP administration for cytokine analysis. For lymph node cell subset analysis, mice were euthanized at 24 h, and inguinal lymph nodes were collected.

### Flow cytometric analysis of cell subsets in the draining lymph nodes

Inguinal lymph nodes (LNs) obtained from C57BL/6 mice treated with PBS (control), EBOV GP or GP+LPS-RS were placed in 1× PBS and single cell suspensions were generated by mechanical disruption with a syringe plunger and passing through a 70 μm cell strainer. The total number of live cells was calculated by trypan blue exclusion using a hemocytometer. The cells were incubated with anti-mouse CD16/CD32 antibody in staining buffer (1x PBS supplemented with 2% FBS) to minimize non-specific binding, and stained with the following: PE-Texas red-conjugated anti-mouse CD4 (clone GK1.5), APC-conjugated anti-mouse CD8a (clone53-6.7), PerCPCy5.5-conjugated anti-mouse CD11b (clone M1/70), and PE-conjugated anti-mouse CD11c (clone N418). All antibodies were purchased from eBioscience. Different subsets of cells were analyzed on a FACSAria flow cytometer (BD Biosciences) as described previously (Kumar et al., 2013) and the data were analyzed using Flowjo software (TreeStar).

### Measurement of cytokines and chemokines in mice

The levels of multiple cytokines and chemokines in the sera from mice administered with EBOV GP in the presence or absence of LPS-RS were measured using a Bio-Plex Pro™ mouse cytokine standard 23-plex, group I kit (Bio-Rad). Mouse serum samples at 1:3 dilution were assessed for the production of the following cytokines: IL-1α, IL-1β, IL-2, IL-3, IL-4, IL-5, IL-6, IL-9, IL-10, IL-12p40, IL-12p70, IL-13, IL-17A, Eotaxin, G-CSF, GM-CSF, IFNγ, MCP-1, MIP-1α, MIP-1β, RANTES, and TNFα according to manufacturer's instructions. Plates were read using the Luminex 200 xMAP system (Millipore) and data were analyzed using the Luminex xPONENT software (Millipore) as described previously (Kumar et al., 2013). The level of beta interferon (IFN-β) in mouse sera was measured using the VeriKine mouse interferon beta ELISA kit (PBL Assay Science) according to manufacturer's instructions as.

### Statistical analysis

Significant differences in the serum levels of cytokines and chemokines or LN cell subsets between groups of mice were determined by ANOVA tests using GraphPad Prism verson 7.0 (GraphPad software, San Diego, CA). *P* values of <0.05 were considered significant.

## Results

### Immunization with EBOV GP alone induces strong antibody production and affords protection against lethal viral challenge in mice

Mouse models have proven to be useful tools to understand immune responses to filovirus infection and evaluate vaccines and antiviral compounds (Bradfute et al., 2012). Our previous study used a mouse model to evaluate the potential of recombinant EBOV proteins expressed in stably transformed Drosophila S2 cell lines to protect against EBOV infection (Lehrer et al., 2017). We demonstrated that while 3 doses of purified recombinant EBOV GP along with adjuvant completely protected mice challenged with 100 PFU of mouse adapted EBOV, mice immunized with GP alone showed partial protection, with survival of 7 of 10 mice following lethal challenge (Lehrer et al., 2017). To further understand the association between protection and antibody response to EBOV GP alone and GP + adjuvant, we evaluated the kinetics of GP-specific IgG antibodies in BALB/c mice immunized with 10 μg of GP or GP + adjuvant via the subcutaneous route, followed by two booster doses at 4 week intervals post primary immunizations. As seen in Figure 1, all animals immunized with GP + adjuvant demonstrated detectable EBOV-specific antibodies at week 3 after primary immunization that increased sharply after the second dose and remained high after the third dose. In comparison, animals immunized with GP alone developed antibody titers after the first dose which were similar to the GP + adjuvant group, but increased more gradually after the second and third doses. The difference between the GP-specific endpoint IgG titers after the third dose in both groups was not statistically significant. These results suggested that GP alone can elicit potent IgG titers and a protective immune response against EBOV infection.

### EBOV GP is efficiently endocytosed by mouse macrophages and induces upregulation of cytokine mRNA transcription *in vitro*

**Figure 2:**
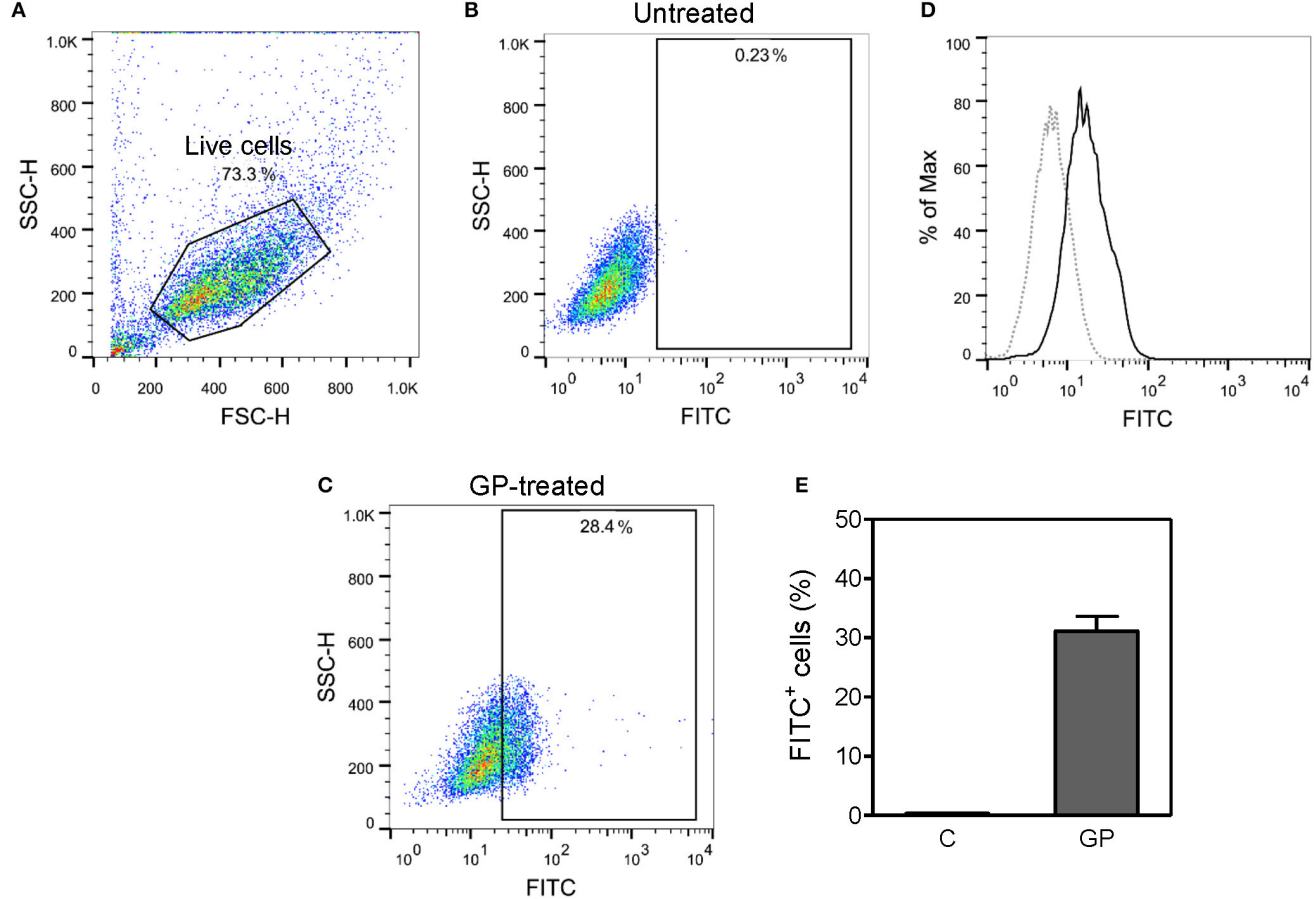
Mouse bone marrow-derived macrophages (BMDMs) can efficiently internalize EBOV GP. BMDMs prepared from C57BL/6 mice were incubated with or without FITC-conjugated EBOV GP (10 μg/mL) at 37°C for 30 min. The cells were washed and fixed, and the antigen uptake was evaluated by flow cytometry. **(A–C)** Live cells were gated based on FSC and SSC, and the percentage of FITC^+^ cells was measured in untreated and GP-treated BMDMs. **(D)** Single parameter histogram of BMDMs incubated with and without GP-FITC. **(E)** Percentage of FITC^+^ BMDMs is expressed as mean ± standard error of mean (SEM) of three independent experiments (C = medium control; GP = GP-FITC).

Since monocytes and macrophages are the initial cell targets of EBOV and the main immune cells that secrete cytokines during EBOV infection (Gupta et al., 2001; Stroher et al., 2001; Hensley et al., 2002), we investigated whether GP can activate these cells and initiate immune responses. As antigen uptake is the first step toward activation of APCs, we determined the ability of BMDMs to internalize GP. The purity of differentiated BMDMs was determined as the percentage of CD11b^+^ cells and was observed to be >95% (data not shown). As shown in Figure 2, GP was efficiently endocytosed by BMDMs and after 30 min of incubation, almost 28–36% of BMDMs were found to be GP-FITC positive.

**Figure 3:**
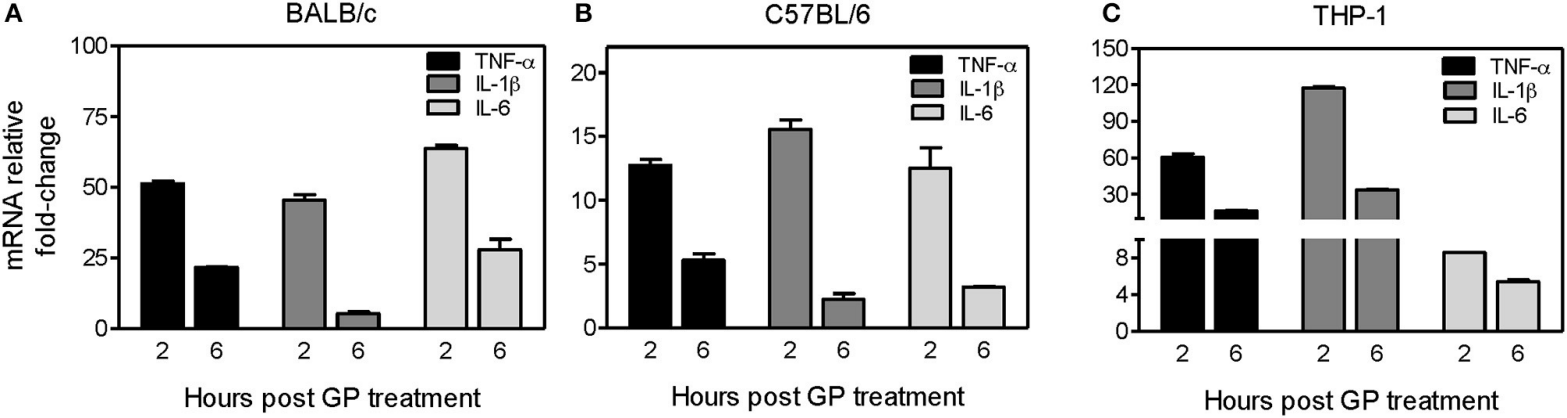
EBOV GP induces gene expression of pro-inflammatory cytokines in mouse and human immune cells. BMDMs prepared from **(A)** BALB/c mice, **(B)** C57BL/6 mice, or **(C)** human THP-1 cells were treated with 1 μg/mL EBOV GP, and total cellular RNA was extracted. The change in the mRNA levels of TNF-α, IL-1β, and IL-6 at 2 and 6 h after treatment was analyzed by qRT-PCR using specific primers. The data was normalized to GAPDH mRNA and fold-change was calculated as described previously. The results are presented as mean ± SEM of at least two independent treatments analyzed in duplicate wells.

We further investigated whether internalization of GP also stimulates the induction of innate immune cytokines in mouse BMDMs. BMDMs derived from both C57BL/6 (Th-1 dominant) and BALB/c (Th-2 dominant) mice were exposed to EBOV GP, and the intracellular levels of mRNA transcripts coding for key inflammatory cytokines were analyzed at 2, 6, and 24 h post-exposure using quantitative real-time PCR. As shown in Figure 3A, pro-inflammatory cytokines TNF-α, IL-1β, and IL-6 were significantly induced (45–64-fold) at 2 h after GP exposure of BALB/c BMDMs, but decreased dramatically at 6 h, and reached basal levels at 24 h post exposure (data not shown). Similarly, GP triggered the transcription of TNF-α, IL-1β, and IL-6 genes in BMDMs of C57BL/6 mice within 2 h of exposure and the levels of the transcribed mRNAs also declined after 6 h of exposure (Figure 3B).

To ascertain whether EBOV GP also induces similar responses in human immune cells, we treated THP-1 cells, a human monocytic leukemia cell line extensively used to study human monocyte/macrophage functions (Chanput et al., 2014), with 1 μg/mL of GP. As shown in Figure 3C, we observed a similar pattern of mRNA transcription of TNF-α, IL-1β, and IL-6 genes in THP-1 cells. TNF-α and IL-1β mRNAs increased by 60 and 120-fold, respectively within 2 h of exposure and declined after 6 h, reaching basal levels comparable to the untreated cells at 24 h after exposure (data not shown). Collectively, these results suggest that EBOV GP can stimulate induction of important innate immune cytokines, which are shown to be essential for the control of viral infection.

### EBOV GP induces the release of innate immune cytokines and chemokines *in vivo*

**Figure 4:**
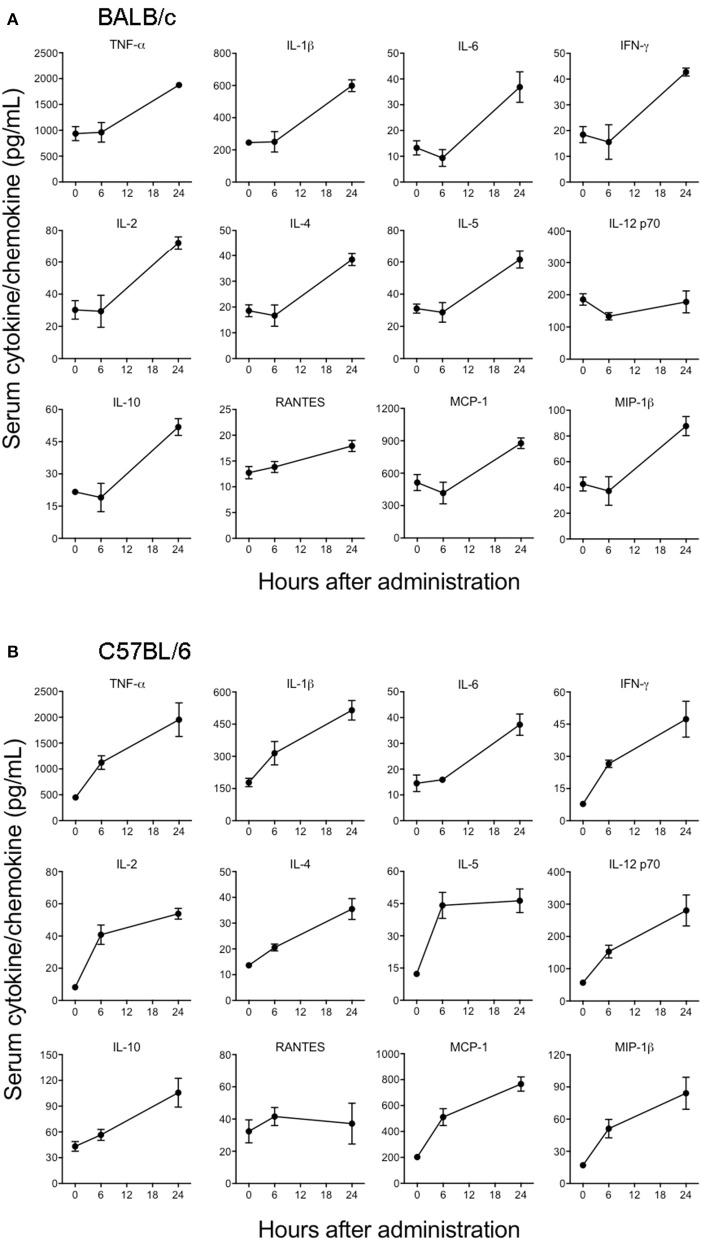
EBOV GP stimulates the production of innate immune cytokines in mice. **(A)** BALB/c and **(B)** C57BL/6 mice were administered EBOV GP (100 μg/mouse) via the i.p. route. Mouse serum was collected at 6 and 24 h after administration. Levels of cytokines TNF-α, IL-1β, IL-6, IL-2, IL-4, IL-5, IFN-γ, IL-12, IL-10, and chemokines MCP-1, MIP-1β, and RANTES were measured using a multiplex Luminex assay. The data are expressed as the mean concentration (pg/mL) ± SEM observed in serum samples from 3 animals per group.

Cytokines and chemokines have been considered to be useful biomarkers for EVD in predicting disease outcome for survivors and non-survivors (Bixler and Goff, 2015). Although previous *in vitro* studies have looked into the innate immune response to EBOV VLPs and shed GP (Wahl-Jensen et al., 2005; Escudero-Perez et al., 2014), the ability of GP to induce cytokines *in vivo* has not been determined so far. Therefore, we next characterized the *in vivo* immune response to GP after administration of 100 μg of EBOV GP to different strains of mice and subsequently analyzed the cytokine levels in serum at multiple time points. As shown in Figure 4A, in GP-treated BALB/c mice we observed significant increases in the levels of key inflammatory cytokines (TNF-α, IL-1β, IL-6), chemokines (MCP-1, MIP-1β), T cell-derived cytokines (IL-2, IL-4, IL-5, IFN-γ), and of the anti-inflammatory cytokine IL-10. RANTES showed only a slight increase at 6 and 24 h while the level of IL-12 did not increase at all. On the other hand, levels of these GP-induced cytokines and chemokines (except for RANTES) were observed to be very high at both 6 and 24 h after EBOV GP treatment of C57BL/6 mice (Figure 4B). We also compared the levels of multiple cytokines induced by GP and the equal amount of protein from the cell culture supernatant from Drosophila S2 cells as NULL control in Swiss Webster mice. Our results demonstrated that while GP induced multiple cytokines and chemokines, their levels in the mice administered with S2 supernatant were comparable with the basal levels observed in control mice injected with PBS thus suggesting that our results were specific to GP (Supplementary Figure 1). Since type I interferon (IFN) production is also an important feature of innate immunity to viruses, we further investigated if GP induces type I IFN *in vivo*. However, we did not observe any change in the levels of IFN- β in the sera of GP-treated mice as compared to controls (Supplementary Figure 2). Collectively, these results provide first *in vivo* evidence that GP is capable of inducing strong innate immune inflammatory responses in different strains of mice.

### EBOV GP triggers the production of inflammatory cytokines through the TLR4 pathway

**Figure 5:**
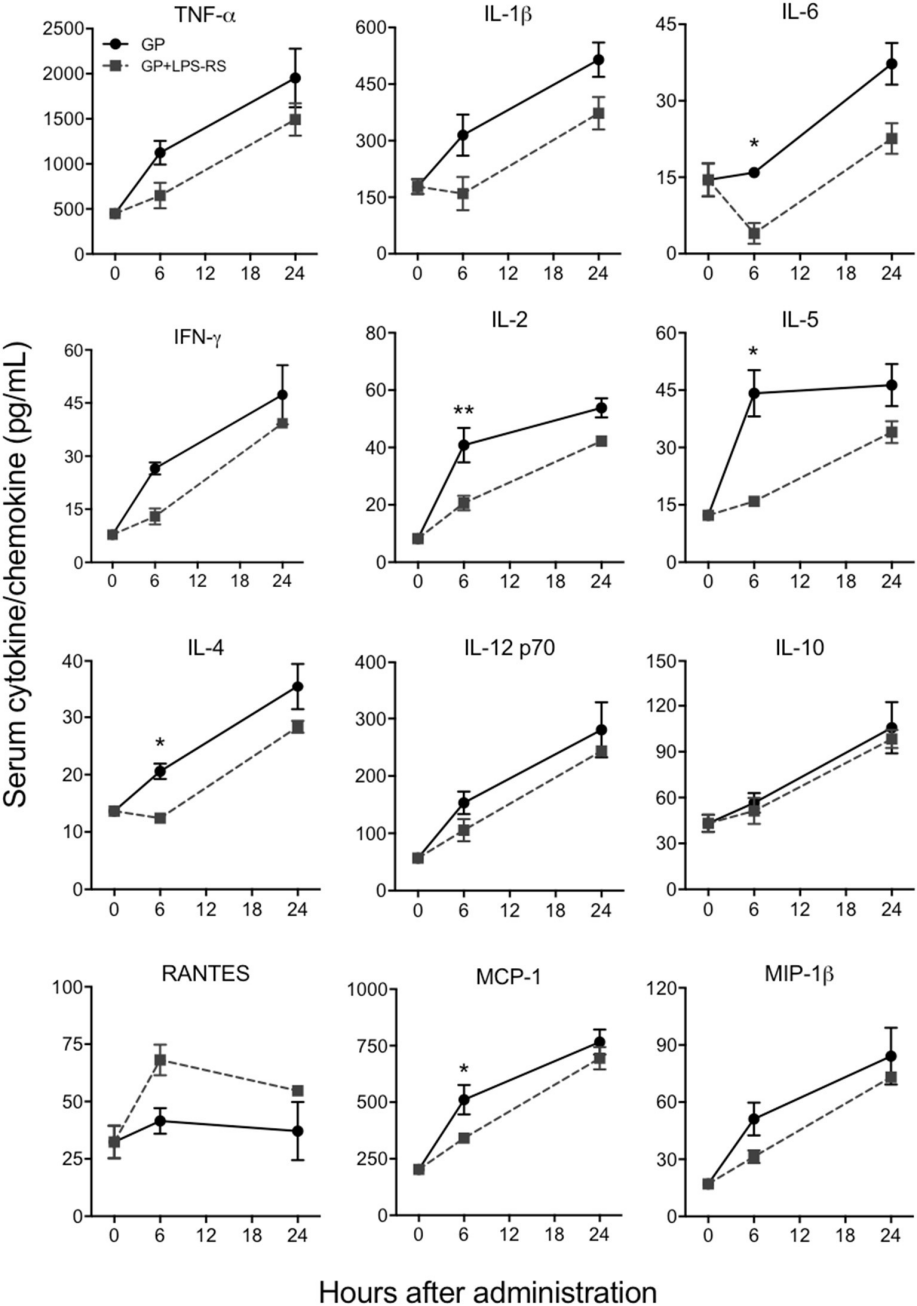
EBOV GP induces innate immune responses via TLR4. C57BL/6 mice were pre-treated with LPS-RS (5 μg per mouse) for 2 days prior to co-administration of 100 μg GP and 10 μg LPS-RS per mouse. Serum levels of multiple cytokines and chemokines were measured using a multiplex Luminex assay. The data are expressed as the mean concentration (pg/mL) ± SEM from at least 3 animals per group. Significance of differences between sera from GP and GP+LPS-treated mice was analyzed by two-way ANOVA followed by a Sidak's multiple comparison test. ^*^p < 0.05, ^**^p < 0.01.

Since TLR4 is proposed to be one of the pathogen recognition receptors that binds to other secretory viral proteins including EBOV shed GP (Ohimain, 2016; Henao-Restrepo et al., 2017), we next tested whether TLR4 also mediates the innate immune responses to purified EBOV GP *in vivo*. The TLR4 signaling pathway was blocked using a commonly used TLR4 antagonist, LPS-RS (Modhiran et al., 2015) and the levels of GP-induced cytokines were compared to a non LPS-RS-treated group. As shown in Figure 5, the levels of key cytokines and chemokines were attenuated in C57BL/6 mice pre-treated with LPS-RS. The differences for IL-6, IL-2, IL-5, IL-4, and chemokine MCP-1 between GP and GP+LPS-RS treated mice were statistically significant at 6 h, while TNF-α, IL-1β, IFN-γ, IL-12, and MIP-1β levels in GP+LPS-RS treated mice also showed a definite trend toward decreased levels as compared to GP treated mice. In contrast, RANTES production was increased by LPS-RS while the induction of the anti-inflammatory cytokine IL-10 was not affected by LPS-RS treatment. Taken together, our data suggest that TLR4 signaling is one of the major pathways involved in the inflammatory response induced by EBOV GP *in vivo*.

### EBOV GP promotes the homing of immune cells to the draining lymph nodes in a TLR4-dependent manner

**Figure 6:**
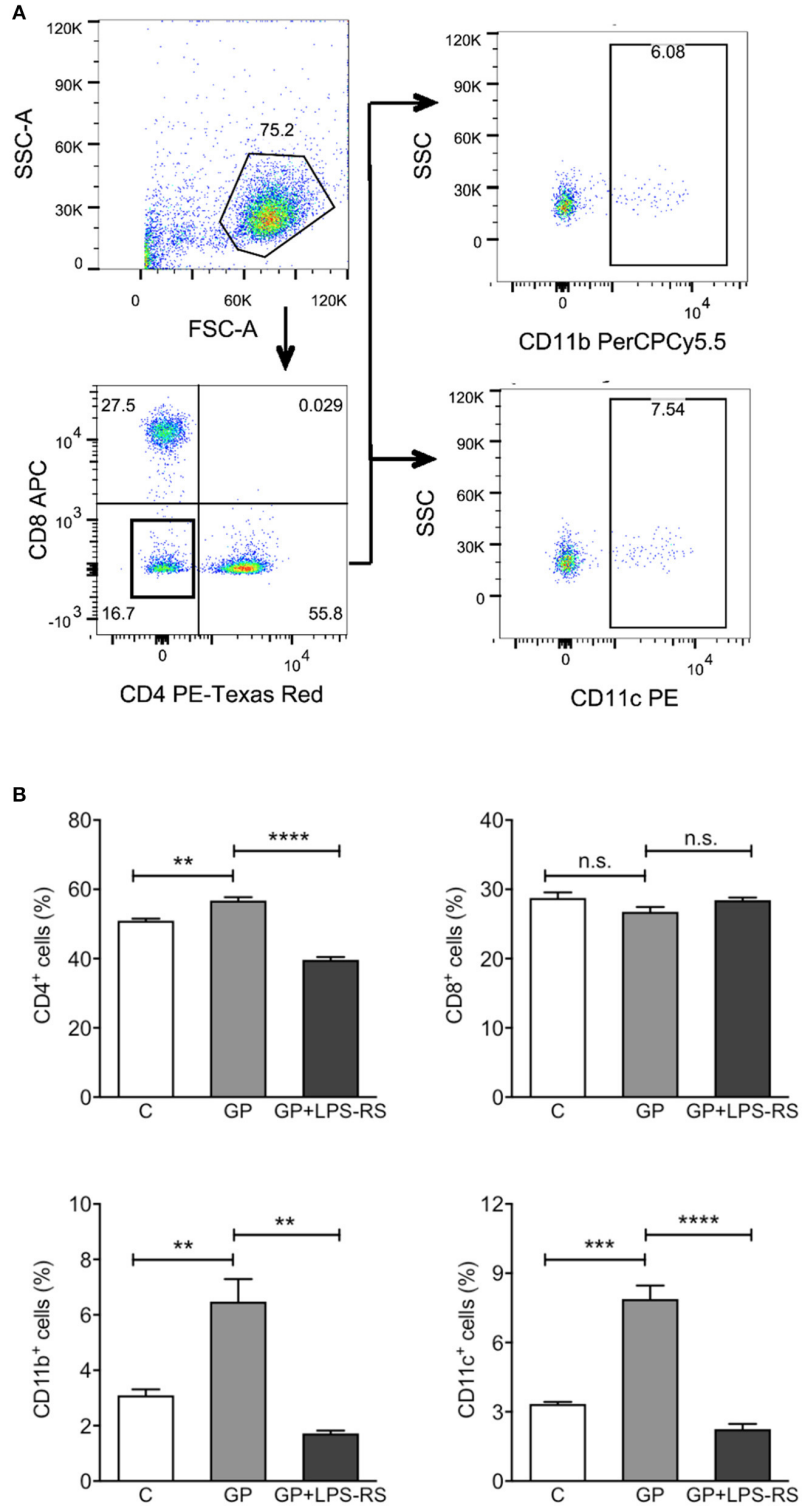
GP-associated innate immune response affects homing of immune cells into the draining lymph nodes. C57BL/6 mice were administered intraperitoneally with PBS (C), GP alone (100 μg per mouse), or GP+LPS-RS as described previously. Inguinal lymph nodes (LNs) were harvested at 24 h after treatment, and disrupted single cell suspensions were stained using fluorochrome-conjugated antibodies specific for CD4^+^, CD8^+^, CD11b^+^, or CD11c^+^ and evaluated by flow cytometry. **(A)** Live cells were determined according to the size and granularity on the FSC vs. SSC histogram, and different subsets of cells were analyzed gated on live cells. CD11b^+^ or CD11c^+^ cells were measured in the cell population that was negative for CD4 and CD8. The figure is representative of three independent experiments. **(B)** The percentages of CD4^+^, CD8^+^, CD11b^+^, and CD11c^+^ cells in the LNs of control, GP or GP+LPS-RS-treated mice are expressed as mean ± SEM of three independent experiments in flow cytometry (*n* = 3 per group). Significance of differences between treatments was analyzed by one-way ANOVA followed by a Tukey's multiple comparison test. ^**^p < 0.01, ^***^p < 0.001, ^****^p < 0.0001.

After vaccination or in the natural course of infection, production of cytokines and chemokines leads to the recruitment of activated APCs to the DLNs, where they activate the proliferation of antigen-specific T cells (De Gregorio et al., 2013). Given that GP induced cytokines and chemokines *in vivo*, we next assessed whether GP also affects the homing of immune cells to DLNs. Single cell suspensions prepared from inguinal LNs of mice treated with EBOV GP were used to evaluate different cell subsets using flow cytometry. CD11b^+^ macrophages and CD11c^+^ DCs were analyzed after the exclusion of CD4^+^ and CD8^+^ cells. As shown in Figure 6, in comparison to the mock group, significantly increased percentages of CD11b^+^ and CD11c^+^ cells were observed in the DLNs after GP treatment. However, pre-treatment of mice with LPS-RS reduced the recruitment of APCs to baseline. The percentage of CD4^+^ cells also increased in the DLNs of GP-treated mice, an effect that was also inhibited by LPS-RS. Collectively, our results suggest that following internalization of GP, activated APCs induced cytokine or chemokine production via TLR4 signaling, which subsequently enhanced the migration of immune cells to the DLNs and may promote the activation and proliferation of CD4 T cells *in vivo*.

### EBOV GP enhances macrophage maturation

**Figure 7:**
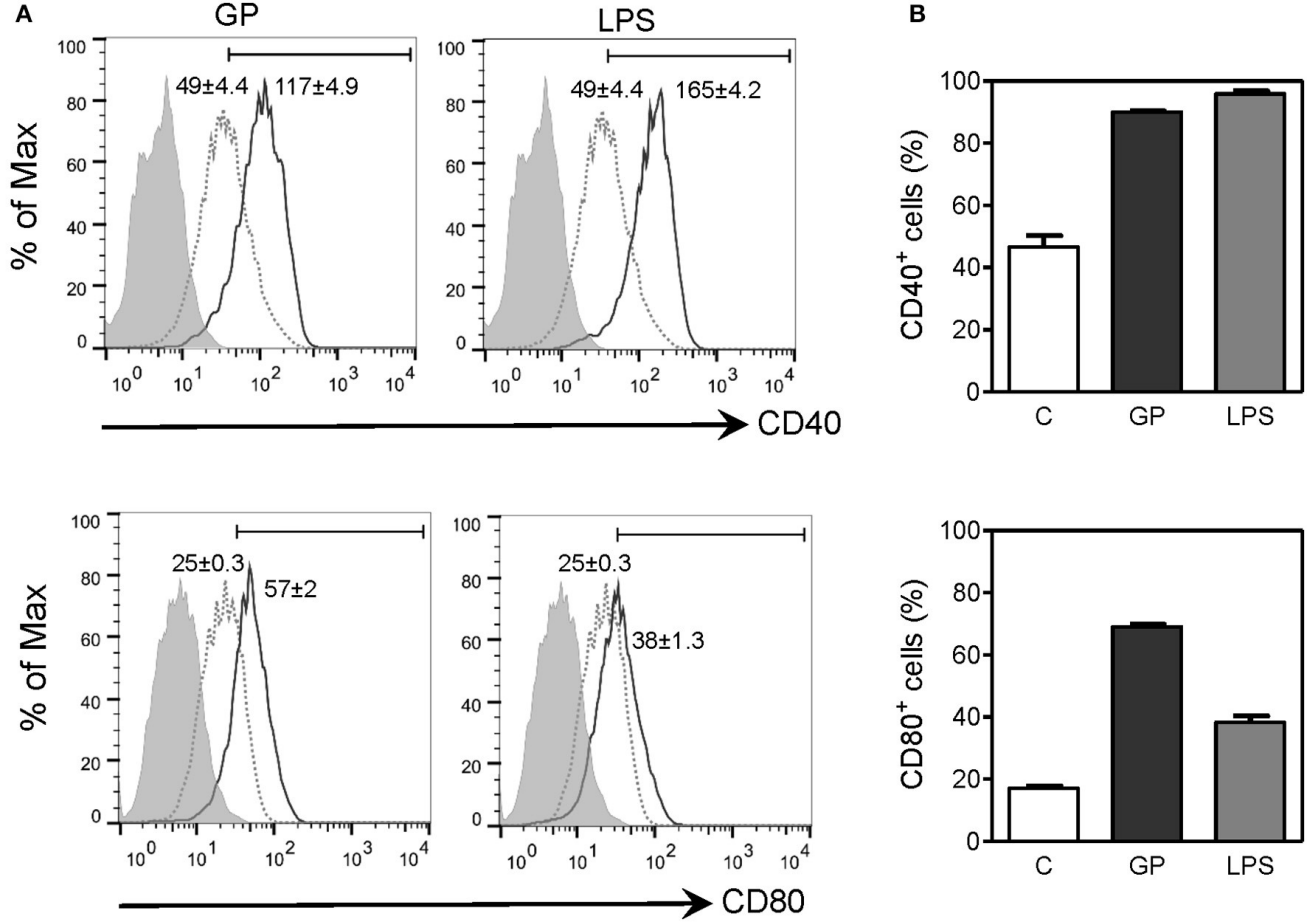
EBOV GP induces the phenotypic maturation of mouse BMDMs. BMDMs from C57BL/6 mice were exposed to 10 μg/mL EBOV GP or 100 ng/mL LPS for 2 days. The cells were washed and stained with fluorochrome-conjugated anti-CD40 and CD80 antibodies, and the surface expression of costimulatory molecules was assessed by flow cytometry. **(A)** One representative is presented in single parameter histograms (gray dotted line: control; black solid line: GP or LPS; gray shade: FMO). MFI (mean fluorescence intensity) values are shown as mean ± SEM of two independent experiments in flow cytometry profiles. Positive cells exhibit a MFI greater than the value of FMO. **(B)** The percentages of CD40^+^ and CD80^+^ cells are expressed as mean ± SEM of duplicate measurements in bar graphs.

Upregulation of costimulatory molecules in APCs is necessary for antigen presentation and priming of T cell responses. Since our results showed that EBOV GP can trigger efficient uptake by macrophages and induce production of inflammatory cytokines, we next assessed whether GP can influence maturation of macrophages, an important event in fine-tuning the innate-adaptive interface. The effect of EBOV GP on the expression of costimulatory molecules CD40 and CD80 on mouse BMDMs was therefore determined by flow cytometry. As shown in Figure 7, in GP-treated macrophages, surface expression of CD40 and CD80 was dramatically increased as compared to control cells, as indicated by the MFI values in flow cytometry profiles and the percentages of CD40^+^ and CD80^+^ cells. The expression levels of CD40 and CD80 in GP-treated BMDMs were comparable to those observed in BMDMs stimulated with LPS as positive control thus suggesting that GP can enhance the ability of APCs to induce T cell activation.

## Discussion

EBOV GP is the major antigen in all Ebola vaccine candidates and has been shown to induce virus-neutralizing antibodies (Takada et al., 2007). EBOV GP expressed in several vaccine approaches using viral vectors or virus-like particles has been shown to protect rodents and non-human primates from EBOV infection (Warfield et al., 2007; Martins et al., 2016). Our previous study showed that highly purified recombinant EBOV GP in combination with matrix proteins VP24, and VP40 with or without adjuvants elicits both effective humoral and cellular immune responses, yielding up to 100% protection in mouse models (Lehrer et al., 2017). Purified proteins alone are generally only weakly immunogenic and need to be combined with adjuvants to enhance T and B cell responses. Interestingly, our previous study also demonstrated that three immunizations with purified recombinant GP alone resulted in 70% protection in mice (Lehrer et al., 2017), suggesting that GP is capable of inducing protective immune responses against EBOV infection. Little is known about the mechanisms by which GP induces protection, however, available data from naturally infected and vaccinated individuals provide indirect evidence for the role of early immune responses in protection against EVD. It has been reported that EVD survivors, as compared to fatally infected patients, develop well-regulated, early, and stronger inflammatory responses, which are proposed to be crucial to control viral replication and induce specific adaptive immunity (Leroy et al., 2000, 2001; Baize et al., 2002). Furthermore, rVSV-ZEBOV, currently the most advanced vaccine candidate, was shown to protect non-human primates even before appropriate adaptive immunity was induced (vaccination 3 and 7 days before EBOV challenge) (Marzi et al., 2015). However, the protection level was reduced when the vaccine candidate was given 20–30 min after viral challenge (Feldmann et al., 2007). A similar trend was observed in the human phase 3 ring vaccination trial in Guinea where it was shown that rVSV-ZEBOV offered substantial protection against EVD from as early as 6 days after vaccination (Henao-Restrepo et al., 2015).

Our current study investigated the early immune responses to EBOV GP and how these responses might affect antigen presentation and the innate-adaptive interface. We demonstrated that highly purified, recombinant EBOV GP was efficiently internalized by macrophages independent of adjuvants that led to the induction of several primary inflammatory cytokines including TNF-a, IL-6, and IL-1β in monocytes and macrophages. *In vivo* mouse studies confirmed that GP treatment increased serum levels of key cytokines and chemokines at early time points. Another important highlight of our data is that GP-induced inflammatory responses *in vivo* are mediated by the TLR4 signaling pathway, and seem to play an important role in the homing of immune cells to the draining lymph nodes. Finally, we showed that the treatment of macrophages with GP triggers expression of key markers of antigen presentation, suggesting that early inflammatory responses induced by GP may further promote the development of an effective adaptive response.

The early stages of EBOV infection, virus entry and fusion, are mediated by surface GP. The transcription of the EBOV GP gene leads to the synthesis of two mRNAs encoding different forms of GPs, the non-structural secreted GP (sGP), and the viral surface GP (Sanchez et al., 1996). The surface GP is composed of two subunits, GP1 and GP2, linked by a disulfide bond (Jeffers et al., 2002), and presents as a trimer on the EBOV surface. During EBOV infection, cleavage of surface GP by the cellular metalloprotease TACE results in the release of truncated surface GP (shed GP) from infected cells (Dolnik et al., 2004). Previous studies have reported that large amounts of sGP and shed GP released from virus-infected cells are detected in the blood of infected patients and Guinea pigs (Sanchez et al., 1996; Dolnik et al., 2004), and have been associated with EBOV pathogenesis. The GP shed from virus-infected cells can bind and activate human dendritic cells and macrophages, leading to the release of pro- and anti-inflammatory cytokines and affecting vascular permeability (Escudero-Perez et al., 2014). In comparison, sGP was not shown to activate macrophages (Wahl-Jensen et al., 2005), suggesting that various forms of GP induce unique immune responses which may lead to different disease outcomes. Based onsize-exclusion chromatography and subsequent analysis of the oligomerization state using EGS-crosslinking, it is clear that the majority of the recombinant GP used in our study resembles the trimeric form of GP presented on viral particles or VLPs and is comparable to the GP produced by virally vectored Ebola vaccine candidates. Our previous study also shows that only N-linked glycosylation sites are processed in the GP1 and GP2 regions our GP protein, a typical observation for glycoproteins expressed in the drosophila expression system.

Our data demonstrated that purified recombinant EBOV GP is capable of activating macrophages indicated by the expression of pro-inflammatory cytokines, TNF-α, IL-6, and IL-1β, which agrees with what was observed in previous studies using VLPs (Bosio et al., 2004; Wahl-Jensen et al., 2005; Okumura et al., 2010; Ayithan et al., 2014). It has been shown that *in vitro*, human macrophages exposed to live Ebola virus or UV-inactivated virus produce high levels of pro-inflammatory cytokines and chemokines (Gupta et al., 2001; Stroher et al., 2001). In studies using non-human primates and guinea pigs, macrophages were also suggested to be one of the major cell targets of EBOV infection (Connolly et al., 1999; Geisbert et al., 2003b,a), and play a key role in dissemination of virus to other tissues. This causes extensive viral replication and tissue damage in multiple organs (Feldmann and Geisbert, 2011; Takada, 2012). The massive release of inflammatory cytokines and chemokines by EBOV infected macrophages has been associated with viral pathogenesis. In EBOV-infected patients, fatal infection was associated with high levels of IL-10 and IL-1RA, modest levels of TNF-α and IL-6, and non-detectable levels of IL-1β, MIP-1α, and MIP-1β, while survivors were characterized by high levels of TNF-α, IL-1β, and IL-6 in plasma (Baize et al., 2002; Wauquier et al., 2010). This striking difference in the key inflammatory cytokines between survivors and non-survivors suggests an association of early innate immune response with EVD outcome. We believe that our results demonstrate balanced cytokine and chemokine responses including high levels of TNF-α, IL-1β, IL-6, MIP-1α, and MCP-1 as well as anti-inflammatory cytokine IL-10 at early time points in GP-treated mice mimicking the pattern observed in EVD survivors.

Our data using LPS-RS provide the first *in vivo* evidence that GP induces the production of pro-inflammatory cytokines and chemokines via TLR4-mediated immune signaling. This agrees with previous *in vitro* studies that reported production of specific pro-inflammatory cytokines by Ebola VLPs containing GP in THP-1 cells and HEK293 cells stably expressing the TLR4/MD2 complex (Okumura et al., 2010). A study recently published by Iampietro and colleagues also provides evidence that EBOV directly binds to CD4+ T cells through GP-TLR4 interaction and furthermore that the resulting blocking of TLR4 results in T cell death (Iampietro et al., 2017). Collectively, our *in vivo* and previous *in vitro* data support the hypothesis that TLR4 is one of the sensors for EBOV GP. Ayithan and colleagues also demonstrated that Ebola VLPs composed of GP, VP40, and nucleoprotein induced the expression of type I IFN and IFN-stimulated genes (ISGs) in murine BMDCs and BMDMs in addition to pro-inflammatory cytokines (Ayithan et al., 2014). However, recombinant EBOV GP in our *in vivo* experiments did not induce IFN-β secretion, suggesting that the induction of type I IFN and ISGs observed in the previous study might have been triggered by viral or cellular proteins other than GP co-purified during the VLP production.

Protein antigens as vaccine candidates are generally less immunogenic than particulate antigens due to size, degradation, non-specific targeting, poor uptake by APCs, and inability to activate APCs (Reddy et al., 2006; Bachmann and Jennings, 2010) thus justifying the use of adjuvants to mediate these responses. In contrast, our findings show that recombinant EBOV GP alone without any adjuvant is able to directly trigger antigen uptake by macrophages and enhance the surface expression of costimulatory molecules CD40 and CD80, which may reduce the threshold necessary for subsequent T cell activation. Another interesting finding of our study is that the migration of activated APCs to the DLNs was TLR4-dependent. The collective response to GP including induction of inflammatory cytokines and APC activation appears to be similar to the mechanisms by which adjuvants enhance vaccine-induced protective immunity (Awate et al., 2013; De Gregorio et al., 2013). For instance, AS04, a licensed adjuvant used in the human papilloma virus (HPV) vaccine Cervarix™, has been shown to induce cytokine response via TLR4 signaling, which leads to optimal maturation of APCs and their migration to the DLNs and activation of antigen-specific T and B cells (Didierlaurent et al., 2009). Based on these studies and our data, we speculate that EBOV GP possesses adjuvant-like properties.

Development of a safe and effective vaccine is important to prevent and combat EBOV infection and two virally vectored EBOV vaccine candidates, rVSV-ZEBOV and adenovirus-based vaccines have proceeded to clinical trials in multiple countries, including some with EVD endemic areas (Martins et al., 2016). In addition, EBOV VLPs containing GP were successful in protecting rodents and non-human primates from EBOV infection (Warfield et al., 2003, 2007; Swenson et al., 2008). Our recombinant EBOV GP possesses a proper trimeric conformation that resembles the GP present on the surface of Ebola virus particles, and most likely also on virally vectored vaccines. We surmise that this is the reason why recombinant EBOV GP is capable of inducing protective immune responses against EBOV. The current study, which is an extension of our previous study (Lehrer et al., 2017), further demonstrates that EBOV GP alone triggers fast, robust, yet balanced innate responses that may play a critical role in the induction of adaptive immunity. The strong early inflammatory responses observed in EVD survivors and rapid protection in individuals who received the rVSV-ZEBOV vaccine highlight the importance of well-regulated innate immune responses in post-exposure protection against EBOV infection. Post-exposure vaccination has been shown to prevent several viral diseases such as rabies, hepatitis B, and small pox in humans (Massoudi et al., 2003; Rupprecht and Gibbons, 2004; Bader and McKinsey, 2013). There is an urgent need to develop effective post-exposure treatments in response to future filovirus outbreaks, to combat bioterrorism, or to treat laboratory exposures. Recent studies reported that VLPs protect mice from EBOV infection when given 24 h post-challenge (Ayithan et al., 2015; Bradfute et al., 2015). VSV-based vaccines given at high dosage levels have also been successfully used in the post-exposure prophylaxis in animals (Feldmann et al., 2007; Geisbert et al., 2008, 2010). However, undesirable reactogenic responses observed in significant numbers of rVSV-ZEBOV vaccinated human subjects when used at high doses (Huttner et al., 2015) raises some concerns with the use of this vaccine in special populations that may also be at risk of acquiring EVD. Our previous and current studies together demonstrated that recombinant EBOV GP can induce appropriate, but confined inflammatory responses and therefore shows a clear safety advantage over virally vectored vaccines. More studies are needed to investigate the effect of GP-induced immune responses in the post-exposure settings. In summary, our data provides *in vivo* mechanistic evidence that recombinant EBOV GP triggers proper innate activation in the absence of adjuvants, which may lead to protection in naïve individuals against EBOV infection. Additionally, the knowledge gained from this study aids in a better understanding of the immunogenicity of EBOV GP and lays a foundation to test its potential use in pre- or post-exposure prophylaxis and for Ebola vaccine development.

## Author contributions

CYL, AL, and SV were involved in conceptualizing the study, experimental design and data analysis. CYL conducted most of the experiments. DS and TW assisted CYL in flow cytometry and animal experiments. CYL wrote the draft of the manuscript that was edited by AL and SV.

### Conflict of interest statement

The authors declare that the research was conducted in the absence of any commercial or financial relationships that could be construed as a potential conflict of interest. The reviewer SY and handling Editor declared their shared affiliation, and the handling Editor states that the process nevertheless met the standards of a fair and objective review
