# Supplementary material for: Ebola Virus Glycoprotein Induces an Innate Immune Response In vivo via TLR4
Source: Front Microbiol. 2017 Aug 17;8:1571. doi: 10.3389/fmicb.2017.01571 (PMC5562721; doi:10.3389/fmicb.2017.01571)
Supplement: Supplementary file 1 [file Presentation1.PDF]

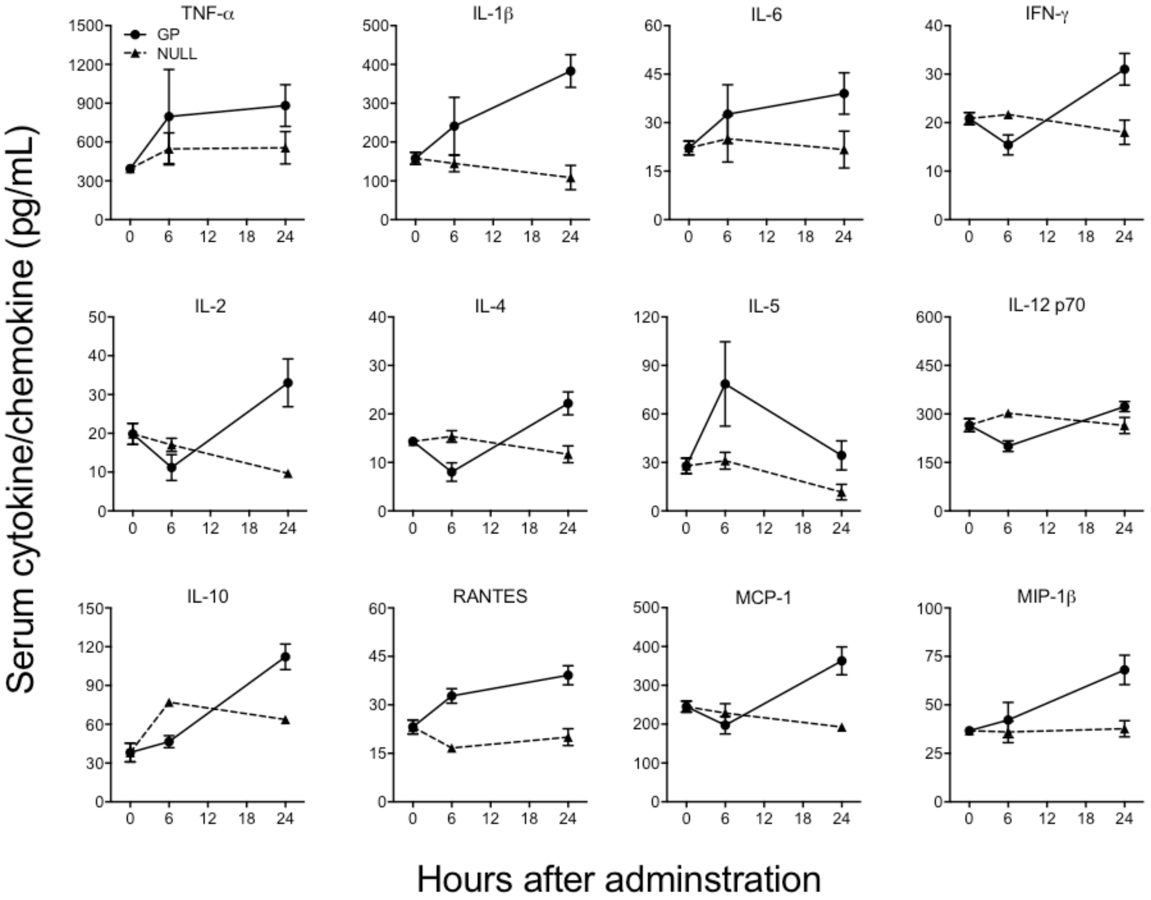

Supplementary Figure 1. Cytokines and chemokines induced by EBOV GP in outbred Swiss Webster mice. Swiss Webster mice were administered with EBOV GP (100 μg/mouse) or equal amount of total protein prepared from the cell culture supernatant of Drosophila S2 cells (NULL control) via the i.p. route. Mouse serum was collected at 6 and 24 hours after administration and levels of cytokines TNF-α, IL-1β, IL-6, IL-2, IL-4, IL-5, IFN-γ, IL-12, IL-10, and chemokines MCP-1, MIP-1β, and RANTES were measured using a multiplex Luminex assay. The data are expressed as the mean concentration (pg/mL) ± SEM in serum samples from 3 to 6 animals per group.

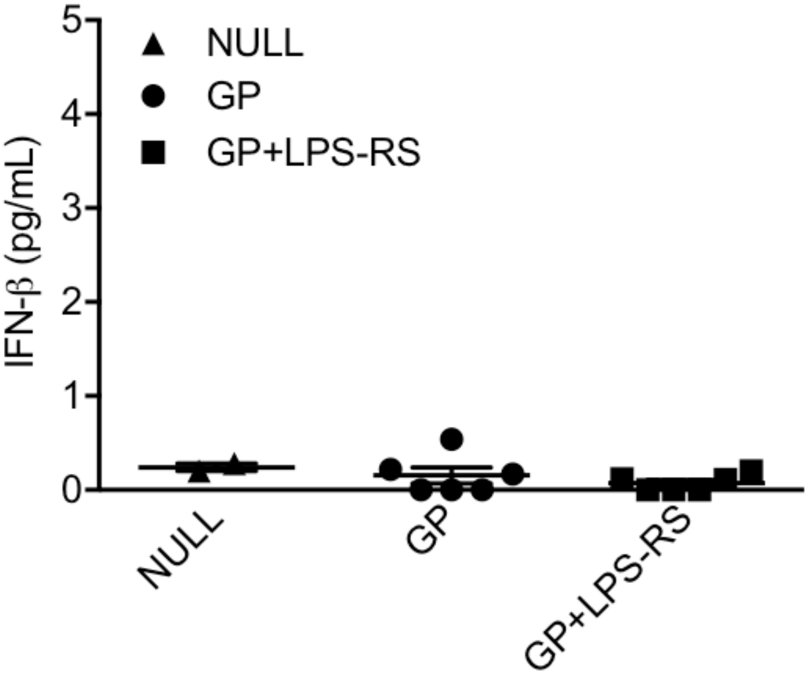

Supplementary Figure 2. The production of type I IFN in mouse serum after treatment with EBOV GP. C57BL/6 mice were administered with GP (100 µg per mouse), GP+LPS-RS or total protein prepared from Drosophila S2 cell culture supernatant (NULL). Mouse serum was collected at 24 hours, and the levels of IFN-β were measured using a commercially available mouse IFN-β serum ELISA kit. The data is expressed as the mean concentration (pg/mL) ± SEM in serum samples from 2 to 6 animals.
